# Catalytic ammonia reforming: alternative routes to net-zero-carbon hydrogen and fuel

**DOI:** 10.1039/d2sc04672e

**Published:** 2022-10-17

**Authors:** Luis C. Caballero, Nicholas E. Thornburg, Michael M. Nigra

**Affiliations:** Department of Chemical Engineering, University of Utah Salt Lake City UT USA Michael.Nigra@utah.edu; Center for Integrated Mobility Sciences, National Renewable Energy Laboratory Golden CO USA Nicholas.Thornburg@nrel.gov

## Abstract

Ammonia is an energy-dense liquid hydrogen carrier and fuel whose accessible dissociation chemistries offer promising alternatives to hydrogen electrolysis, compression and dispensing at scale. Catalytic ammonia reforming has thus emerged as an area of renewed focus within the ammonia and hydrogen energy research & development communities. However, a majority of studies emphasize the discovery of new catalytic materials and their evaluation under idealized laboratory conditions. This Perspective highlights recent advances in ammonia reforming catalysts and their demonstrations in realistic application scenarios. Key knowledge gaps and technical needs for real reformer devices are emphasized and presented alongside enabling catalyst and reaction engineering fundamentals to spur future investigations into catalytic ammonia reforming.

## Abstract 2

Ammonia is a promising net-zero-carbon energy vector whose hydrogen content is accessible *via* multiple catalytic pathways. This perspective highlights advances in NH_3_ reforming catalysis and opportunities for reaction engineering of reformer devices.

## Graphical abstract

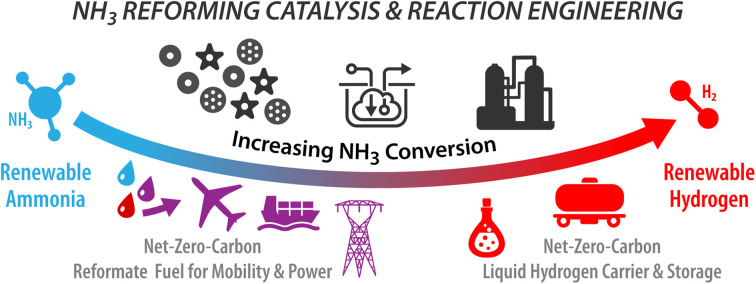

## Introduction

**Scheme 1:**
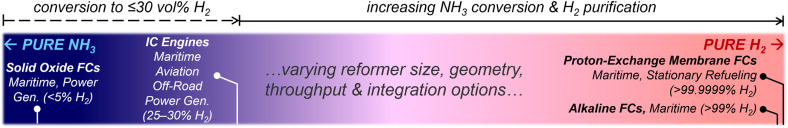
Spectrum of NH_3_-capable internal combustion (IC) and fuel cell (FC) technologies for air, sea and land mobility and for stationary applications as a function of NH_3_ conversion (*i.e.*, H_2_ fraction in reformate). Note that additional hydrogen purification is required following catalytic NH_3_ reforming for FC applications.

Ammonia (NH_3_) is one of the most critical industrial chemicals for sustaining global life, with around 180 million t produced globally every year exclusively *via* Haber–Bosch-type routes.^1^ In addition to its current major uses as a fertilizer, chemical intermediate and refrigerant, ammonia is poised to play a crucial role in substituting conventional fossil fuels and hydrogen (H_2_) resources for future energy and chemical platforms. The U.S. Department of Energy (DOE) has recently identified NH_3_ as a promising H_2_ carrier for advanced mobility and storage,^2^ owing to its high active hydrogen content (17.8 wt%), ease of liquefaction and accessible dissociation chemistries. Although pure NH_3_ has poor fuel properties itself,^3^ blended or reformed mixtures of H_2_ and NH_3_ are higher performing in internal combustion (IC) engines.^4,5^ Emerging, renewable-electron-driven NH_3_ synthesis pathways^6,7^ enable the molecule's prospect as a net-zero-carbon (NZC) H_2_ carrier and/or fuel for advanced IC piston and turbine engines and for fuel cells (FCs), broadly applicable to each transportation and stationary energy applications (Scheme 1).

Ammonia reforming (*i.e.*, ammonia dissociation or decomposition) occurs in the presence of a heterogeneous catalyst to produce hydrogen, nitrogen (N_2_) and possibly water (H_2_O) if oxygen (O_2_) is present in the feed gas. The extent of NH_3_ conversion is a strong function of catalyst identity, reforming reaction conditions, participating reforming pathways, reactor geometry, fluid contacting patterns, transport phenomena and more, and different NH_3_ reforming applications dictate unique performance targets and design constraints. Generally, air-,^8,9^ sea-^10,11^ or land-borne^12,13^ vehicles utilizing ammonia as a liquid fuel and/or carrier will require on-board catalytic reforming reactors, akin to hydrocarbon fuel reforming strategies,^14,15^ whereas stationary applications for hydrogen storage and dispensing may have lesser spatial constraints but much stricter hydrogen product purity specifications; in particular, ultra-high H_2_ purities are required for NH_3_/H_2_ FC converters when NH_3_ is used as the carrier^16^ (Scheme 1). However, unlike high-pressure hydrogen, well-established NH_3_ infrastructure and supply chains will sustain rapid adoption of new customer end-markets while renewable NH_3_ generation technologies mature to scale. Ultimately, the future viability of ammonia as a NZC H_2_ carrier or fuel for IC engines, FC systems and stationary hubs—by air, sea or land—hinges upon the development of efficient, highly integrated catalytic reforming reactors to generate requisite hydrogen energy or fuel at tunable purities for a given end use.

In this Perspective, we review recent advances in heterogeneous catalysis for ammonia reforming alongside enabling reaction engineering principles to motivate future research investigations into this important route to NZC hydrogen and fuel for difficult-to-decarbonize modes of global transportation. We draw parallels across various time and length scales of NH_3_ reforming to illustrate the unique multidimensionality of this problem, as well as the hurdles that belie its graduation from laboratory to commercial use. While H_2_O electrolysis and renewable NH_3_ synthesis systems continue to mature, here we identify key knowledge gaps in catalytic ammonia reforming strategies and technologies to prescribe actionable goals for fundamental researchers and industrial practitioners alike. Successful advances in ammonia reforming will help bridge critical deployment barriers in hydrogen-at-scale, offering flexible, alternative pathways to these NZC energy vectors.

## Fundamentals of ammonia reforming catalysis

Catalytic routes to ammonia reforming provide potentially lower pressure and temperature pathways to non-electrolytic hydrogen production. As the reverse reaction of ammonia synthesis, ammonia decomposition is an endothermic reaction, and temperatures of 400 °C are needed to drive the reaction to >99% equilibrium conversion of ammonia. However, reforming applications for IC engine feeds will likely require high-pressure operating conditions that adversely shift equilibrium in favor of the reactants, requiring a new understanding of catalysts in these unfavorable environments.

In this section, we will present different catalytic pathways for ammonia reforming focusing on (1) thermal and photocatalytic reforming (*i.e.*, dissociation; eqn (1)), and (2) oxidative reforming (eqn (2)). This Perspective will emphasize thermal and oxidative reforming and provide a brief introduction to photocatalytic reforming. Electrochemical ammonia reforming and ammonia combustion (eqn (3)) will not be discussed. We further note that the terms “reforming”, “dissociation” and “decomposition” may be used interchangeably in the context of these ammonia reactions; however, “cracking” is an inappropriate term to describe the chemistry of this small molecule (just as for methane (CH_4_)), regardless of pathway or driving force, and should instead be reserved for long-chain hydrocarbon conversion processes.

*Thermal reforming*

1 NH_3(g)_ ⇆ 0.5 N_2(g)_ + 1.5 H_2(g)_, Δ*H*° = +46 kJ mol_NH_3__^−1^

*Oxidative reforming*

2 NH_3(g)_ + 0.25 O_2(g)_ → 0.5 N_2(g)_ + H_2(g)_ + 0.5 H_2_O_(g)_, Δ*H*° = −75 kJ mol_NH_3__^−1^

*Stoichiometric combustion*

3 NH_3(g)_ + 0.75 O_2(g)_ → 0.5 N_2(g)_ + 1.5 H_2_O_(g)_, Δ*H*° = −317 kJ mol_NH_3__^−1^

The next three subsections describe thermal reforming, oxidative reforming and photocatalytic reforming catalysts to highlight the current states-of-art as well as historical perspectives in each area. Opportunities for future research directions will also be presented.

### Thermal reforming catalysis

#### Mechanistic studies

In 1980, Ertl *et al.*^17^ studied the mechanism of NH_3_ decomposition (eqn (1)) on iron surfaces, in which the adsorption of NH_3_ was proposed to initiate the surface reaction. The sequential cleavage of N–H bonds follows this initial step to produce bound –H atoms, which in turn combine on the surface to form hydrogen molecules that subsequently desorb. A final step consists of the recombination and desorption of two adsorbed –N atoms as molecular nitrogen. Two of these processes have been hypothesized as rate-determining steps (RDSs): ammonia adsorption and nitrogen desorption. Takezawa *et al.*^18^ used a synthetic iron catalyst composed of 4.72% Al_2_O_3_, 0.31% K_2_O, and 0.05% SiO_2_ to show that the RDS of the reaction changed according to temperature. Evidence of nitrogen inhibition is found in the observed reaction rate at temperatures above 479 °C. This supports the hypothesis that two different steps dominate the reaction as a function of temperature: nitrogen desorption dominates at lower temperatures, whereas dehydrogenation of adsorbed amino radical ˙NH_2_ species dominates at higher temperatures. Similarly, a study published by McCabe^19^ showed that the NH_3_ decomposition mechanism over nickel wires transitions above 1000 K, wherein first-order kinetics become dominant and apparent activation energy decreases, corresponding to rate control by the ammonia adsorption step. In contrast, nitrogen desorption was postulated to dominate in the zero-order regime at lower temperatures.

Tamaru^20^ applied a dynamic approach to the study of NH_3_ decomposition kinetics by elucidating its mechanism over different metal-based catalyst surfaces in 1988. The study first examined the reaction over a tungsten catalyst, determining that the overall reaction order was unity with respect to NH_3_ at lower ammonia pressures, approaching zero at higher ones. He further concluded that the rate is always zero-order with respect to H_2_ pressure for this reaction pathway over tungsten. However, mechanistic behavior varies significantly over other transition metals such as iron, where faster hydrogenation of chemisorbed nitrogen dominates. This case typically occurs at lower temperatures and is referred to as the Temkin–Pyzhev mechanism. Conversely, if desorption of nitrogen occurs more rapidly, which is the case at higher temperatures, the tungsten-type behavior is observed. Therefore, the mechanistic pathway depends on the operating temperature, the partial pressure of the reactants and the identity of the catalyst, as stated by Löffler *et al.*^21^ in 1976.

#### Selection of metals

**Fig. 1:**
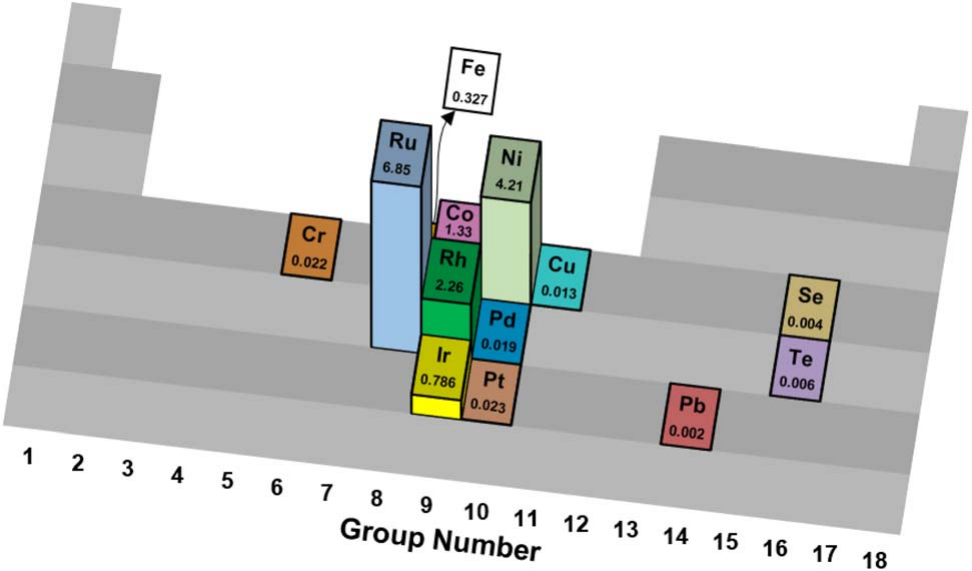
Turnover frequencies (s^−1^) of various catalytic elements supported on Al_2_O_3_ for thermal reforming of NH_3_.^35^

Development of new active catalyst phases and supports have been investigated for thermal reforming of ammonia. The primary metals and alloys that have been studied are Fe,^22–24^ Ni,^25–28^ and Ru.^29–31^ Additionally, Novell-Leruth *et al.*^32^ studied this reaction on the surfaces of platinum-group metals Pd, Rh and Pt, wherein Rh was the most active and exhibited the lowest activation barrier. Recent progress using other metals as active phases, including novel bimetallic compounds containing Co, Mo, Fe, and S, represent a promising step towards highly active and more Earth-abundant reforming catalysts. In more recent work, high-entropy alloys (HEAs) are proposed to enhance activity and stability under elevated temperatures.^33,34^ As a large comparison study, Ganley *et al.*^35^ examined thirteen catalytic materials supported on Al_2_O_3_ for NH_3_ decomposition. They observed that the NH_3_ oxidation activity at 580 °C of metals supported on Al_2_O_3_ followed this trend: Ru > Ni > Rh > Co > Ir > Fe ≫ Pt > Cr > Pd > Cu ≫ Te, Se, and Pb (Fig. 1). No distinct periodic trends were apparent from this investigation. Nitrogen desorption is the RDS on Fe, Co, and Ni; in contrast, on all the other metals, N–H bond breaking is rate-determining. The results from this study corroborate data found in the literature, as Ru has been regarded as the most active metal for thermal reforming of NH_3_ and Ni is reported as the best performing among non-noble metal catalysts.^36–38^

The differences in mechanistic pathways depend on the metal that is used, and the wide range of reaction conditions result in these active metals having clear advantages over one another in particular situations. Ru-based catalysts exhibit high catalytic activity. Supports play a crucial role in these materials, with carbon nanotubes (CNTs),^39^ MgO,^40^ and MgAl_2_O_4_ (ref. 41) showcasing different catalytic activities. Similarly, different promoters such as Cs, K and Na have been studied with varying results.^42^ Ru is the most promising metal for ammonia decomposition on the basis of reaction rate. Nonetheless, its major drawback is its higher price compared to Fe and Ni, which serves as a barrier to industrial applications.

Iron-based catalysts are used for ammonia decomposition in hot gas from coal gasification. Fe catalysts have lower activity than Ru catalysts. However, these materials provide valuable insight into the kinetics of the reaction and the interactions with support materials. Some of the studied support materials include CNTs and mesoporous carbon, such as CMK-3. These Fe catalysts are often compared to Ni-based catalysts, which have niche applications in microstructured reactors for hydrogen production. They provide an attractive alternative, as Ni has a lower cost than Ru and displays higher activity than Fe.^43^ Despite these advantages, ammonia decomposition with Ni as the active metal is highly dependent on structural parameters, such as particle size.^44^ Such considerations also apply to cobalt-based catalysts. Their activity is a strong function of the physical and chemical properties of the support material.^45^ Co-based catalysts have become relevant for ammonia decomposition due to their low cost compared to other active components. Metals such as Pt and Rh have also been assessed for their feasibility. Pt-based catalysts have significant drawbacks, as they must be part of a bimetallic system. Pt is not a very active catalyst for ammonia decomposition by itself, as conversions of only 2% are achieved with monometallic Pt catalysts.^39^ Its advantages lie in its role as an activity enhancer when combined with other metals such as Ni, Fe, or Co.^46^

Table 1 summarizes results across different studies of catalytic materials for NH_3_ decomposition. Taken together, these results corroborate Ru being an effective and frequently studied catalytic metal for NH_3_ thermal reforming.

**Table 1:** Catalytic activity, reaction conditions and performance metrics for NH_3_ decomposition catalysts reported in literature.^a^^,^^b^

Catalytic metal	Metal content (wt%)	Promoter	Metal : promoter (mol mol^−1^)	Support material	Temperature (°C)	Pressure^b^ (atm)	GHSV (mL g_cat_^−1^ h^−1^)	TOF (min^−1^)	NH_3_ Conversion (%)	Ref.
Ru	3.0	—	—	Ba–ZrO_2_	450	—	30 000	26.6	23.6	49
Ru	3.0	K	2 : 1	Ba–ZrO_2_	450	—	30 000	36.7	32.5	49
Ru	3.0	Cs	2 : 1	Ba–ZrO_2_	450	—	30 000	42.8	37.8	49
Ru	2.5	—	—	β-SiC	400	1	60 000	8.4	99.3	50
Ru	5.0	—	—	MgO	450	—	60 000	21.0	30.9	51
Ru	5.0	—	—	Al_2_O_3_	450	—	60 000	15.8	23.4	51
Ru	5.0	K	1 : 1	Carbon nanotubes (CNTs)	450	—	60 000	65.9	97.3	51
Ru	5.0	—	—	CNTs	550	1	30 000	52.6	84.7	52
Ru	5.0	—	—	Graphitic carbon (GC)	550	1	30 000	58.8	95.0	52
Ru	3.2	—	—	CNFs	500	1	6500	22.7	99.0	53
Ru	11.7	K	1 : 5	Graphene aerogel (GA)	450	1	30 000	25.9	97.6	54
Ru	5.0	—	—	Cr_2_O_3_	600	—	30 000	62.1	100	55
Ru	4.8	—	—	La_2_O_3_	525	1	18 000	25.6	90.7	56
Co	10.0	—	—	Multi-wall CNTs	500	1	6000	3.0	74.6	57
Fe	1.29	—	—	GC	600	—	6000	20.6	71.0	58
Ir	10.0	—	—	SiO_2_	550	—	30 000	18.3	30.4	59
Ni	10.0	—	—	SiO_2_	550	—	30 000	4.0	21.6	59
Ni	5.0	K	1 : 2	SiO_2_ (fumed)	550	—	30 000	16.8	42.6	60
Ni	15.0	—	—	MgAl (6 : 1)	550	—	30 000	6.3	48.0	61
Ni	10.0	—	—	La_2_O_3_	550	1	30 000	1.6	59.0	62
Rh	4.9	—	—	CNTs	400	—	30 000	9.0	3.0	39
Pt	9.3	—	—	CNTs	400	—	30 000	2.5	2.0	39
CoMo	5.0	—	—	γ-Al_2_O_3_	600	—	36 000	82.7	99.5	63
CoFe_5_	5.0	—	—	CNTs	600	—	36 000	90.2	48.0	64
MoS_2_	6.0	—	—	Laponite	600	—	24 000	25.9	35.0	65

a All reactions were performed in a fixed-bed flow reactor.

b Pressure conditions assumed to be atmospheric if not explicitly stated.

#### Influence of catalyst preparation method

In evaluating different materials, the synthesis method and final metal loading must be considered. Lorenzut *et al.*^47^ supported Ru nanoparticles by embedding it within a lanthanum-stabilized zirconia (LSZ), a technique hypothesized to mitigate Ru sintering and improve conversion relative to conventional impregnation synthesis methods. Similarly, Hu *et al.*^48^ synthesized a series of supported Ni/ZSM-5 catalysts and showed that metal–support interactions are essential for catalytic activity. Modified solid-state ion exchange (MSSIE) was found to exhibit the highest activity out of all synthesis methods, including wet impregnation (IM), deposition–precipitation (DP) and solid-state ion exchange (SSIE). The 5% Ni/ZSM-5 catalyst synthesized by MSSIE exhibited an NH_3_ conversion of 97.6% at 650 °C. The other synthesis methods led to conversions of 50.1% for IM, 81.3% for DP and 92.9% for SSIE. Earth-abundant supports such as red mud^66,67^ and attapulgite clay^68^ have been considered in metal-based catalysts to minimize environmental impact. Hu *et al.*^69^ investigated Ni supported on mica, a natural silicate mineral with high thermal stability and large porosity, achieving NH_3_ conversion of 97.2% at 15 wt% Ni. Researchers are recommended to pursue new catalyst formulations and synthesis methods that minimize the environmental impact of material preparation while improving activity and time-on-stream stability.

#### Catalyst stability

Studies by Lorenzut *et al.*,^47^ Yin *et al.*^51^ and Zhang *et al.*^70^ assessed catalyst stability for different materials and provided insight into several factors influencing catalytic deactivation, including sintering, synthesis methods and support types. Inokawa *et al.*^36^ studied the thermal stability of Ni nanoparticles synthesized in zeolite pores by a method that they had developed previously.^71^ This approach was based on the adsorption and decomposition of a sublimated Ni organometallic compound. This catalyst was stable up to 500 °C. They attributed these promising results to the micropores present in the structure, which prevented the diffusion and sintering of the nanoparticles. The catalytic support plays a crucial role in its stability, as Huang *et al.*^56^ showed in their 2019 study. They synthesized Ru-based catalysts supported on La_2_O_3_ and compared them with Ru/C catalysts. When compared to carbon-supported catalysts at high temperatures, carbon supports in hydrogen atmospheres are prone to inevitable methanation reactions, thus leading to deactivation. They found their La_2_O_3_-supported catalyst to be more stable without sacrificing catalytic performance. They attributed the higher thermal stability of the catalyst to the spatial isolation and dispersion of the Ru nanoparticles by the support. A different study by Wu *et al.*^72^ investigated a bimetallic Ni–Co catalyst supported on fumed SiO_2_. They evaluated the material's stability under a GHSV of 30 000 mL h^−1^ g_cat_^−1^ for 30 hours and found only a minor decrease in NH_3_ conversion, denoting significant thermal stability. Such developments are pivotal in understanding the importance of designing and synthesizing stable catalysts for the thermal reforming of NH_3_ for large-scale applications.

#### Amides as catalysts

Amides also can serve as NH_3_ decomposition catalysts. David *et al.*^73^ tested sodium amide as a catalyst and achieved 99.2% conversion of NH_3_. Others have also tested lithium amide and potassium amide catalysts with successful results.^74–77^ Mechanistically, it is hypothesized that the alkali amide decomposes to the solid alkali metal, nitrogen and hydrogen gas, and then the solid alkali metal reacts with ammonia to reform the alkali amide and hydrogen.

### Oxidative reforming catalysis

Oxidative reforming (eqn (2)) provides an alternative, exothermic pathway for ammonia decomposition. In contrast to combustion (eqn (3)), here a sub-stoichiometric amount of O_2_ is co-fed to the reactor with NH_3_ reactant. The catalyst bed is heated through the exothermic combustion of ammonia (−200 kJ mol_Ru_^−1^).^78^

There are a number of important material property considerations when designing an oxidative reforming catalyst: (1) active phase composition, (2) support material, (3) promoters, (4) basicity/acidity, (5) surface area and (6) low amount of electron-withdrawing groups.^79^ The RDS may be different depending on the composition of the active phase. For example, dehydrogenation of NH_3_ is rate-determining on noble metal catalysts, whereas the associative desorption of –N atoms is the rate-determining step on non-noble metal catalysts. Further, it is important to choose a catalytic material which does not adsorb CO_2_ and H_2_O very well; otherwise, a catalyst pre-treatment step will be necessary. CO_2_ and H_2_O may out-compete NH_3_ and O_2_ for binding sites on the catalyst, but adsorption behaviors may be mitigated through rational design.

Theory suggests that iridium can produce N_2_ with 100% selectivity from NH_3_.^80^ While environmentally acceptable as a catalytic metal, a main drawback of Ir is its high cost,^81^ which has motivated continued exploration of ruthenium-based catalysts for oxidative reforming of NH_3_. It has been proposed that adsorbed ammonia reacts with adsorbed oxygen atom or hydroxyl groups on the active metal surface to remove a hydrogen from ammonia. It is also hypothesized that ammonia dissociation is dependent on co-adsorbed oxygen. The adsorbed oxygen or hydroxyl group also reacts with NH to remove its hydrogen. Co-adsorbed oxygen increases the ammonia desorption on Ir surfaces.^80^ There are still opportunities for additional studies for further study into the mechanism on Ir and other catalytically active metals. Ru also suffers from deactivation. While the mechanism for deactivation has not been thoroughly studied, it is hypothesized that it is caused by oxide formation, nanoparticle sintering, and diffusion of Ru into the support.^82^ One of the first studies with Ru/Al_2_O_3_ catalysts for oxidative reforming was published in 1967 by Schriber and Parravano with Ru nanoparticles.^83^ They observed that the rate was dependent on the partial pressures of NH_3_, O_2_ and H_2_O. Higher H_2_O partial pressures decreased the NH_3_ oxidation rate, while higher O_2_ partial pressures increased the NH_3_ oxidation rate.

**Fig. 2:**
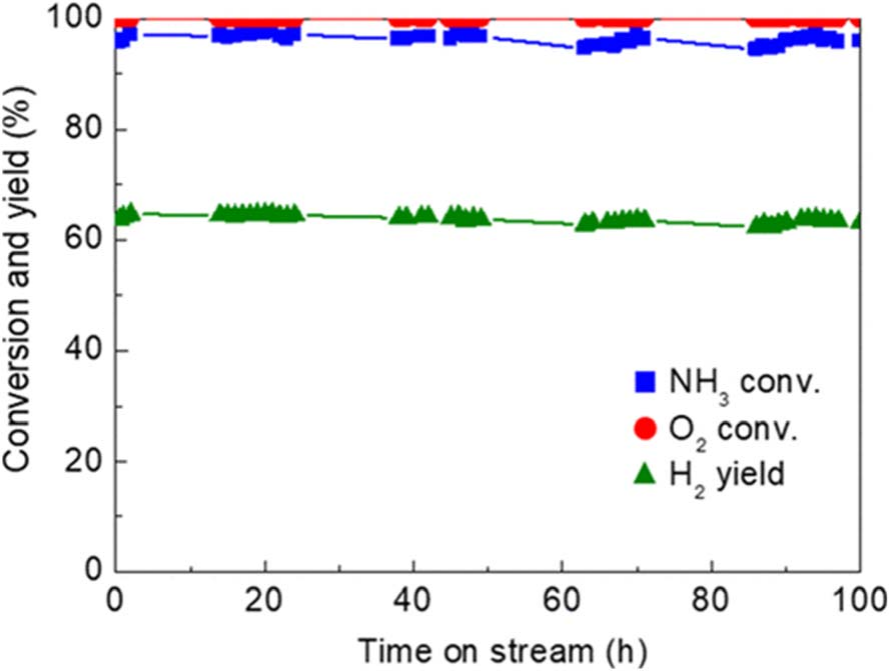
Time-on-stream activity and stability of Ru/Ce_0.5_Zr_0.5_0_2_ catalyst (1 wt% Ru loading) for oxidative reforming of NH_3_.^85^ Reaction conditions : Feed rate 150 : 37.5 : 20.8 (NH_3_ : O_2_ : He) mL min^−1^, 62.5 L h^−1^ g^−1^ space velocity, quasi-adiabatic operation at ambient initial temperature. Reproduced with publisher permission from ref. 85.

One of the recent successful demonstrations of oxidative reforming of NH_3_ was reported by Nagaoka *et al.*^84^ using Ru oxide nanoparticles supported on γ-Al_2_O_3_ and La_2_O_3_. The catalyst is pre-treated with He to remove CO_2_ and H_2_O from the surface, which forms Lewis acid sites *in situ* to activate NH_3_. The heat produced from ammonia adsorption and oxidation can in turn be used to drive NH_3_ decomposition, allowing the reaction to proceed autothermally. In this manner, Ru oxide/γ-Al_2_O_3_ is a more active catalyst than Ru oxide/La_2_O_3_. The hypothesis for this difference in activity is that La_2_O_3_ does not have the Lewis acid sites needed for NH_3_ adsorption. The quantity of NH_3_ adsorbed onto the γ-Al_2_O_3_-supported sample was 8× higher than that of the La_2_O_3_-supported catalyst, contributing to the former support's higher performance within this proof-of-concept study. Another noteworthy example of Ru catalytic activity of NH_3_ oxidative decomposition is Ru/Ce_0.5_Zr_0.5_O_2−*x*_ by Matsunaga *et al.*^85^ Conversion of NH_3_ was reported to be >96%, with N_2_ and H_2_ yields of >96% and 63%, respectively; oxygen conversions were seen to be virtually 100% for this catalyst (Fig. 2). NH_3_ conversion increased with decreasing NH_3_/O_2_ feed ratios. Ru clusters have been immobilized on alkali-exchanged Y zeolites by Cha *et al.*^86^ Of the catalysts prepared, Ru/Rb–Y was most active for oxidative reforming, attributed to the low acidity of the Rb–Y and higher electron density around the Ru sites, which allows for more facile N_2_ desorption.

There have been advances in alloying Ru with other metals to enhance the aggregate catalyst's performance. Experiments performed by Chakraborty *et al.*^87^ combined Cu with Ru to study their performance for oxidative reforming at 170 °C under ultra-high vacuum (UHV) conditions. The two metals are immiscible, and the Cu naturally segregates to the surface of the nanoparticles. Ammonia consumption rate enhancements were observed to be 3× greater with respect to the monometallic Ru catalysts and 40× with respect to monometallic Cu catalysts, with the best bimetallic compositions determined to be 0.6–0.8 monolayers of Cu on Ru. All bimetallic compositions exhibited higher activities than the monometallic Cu and Ru.

Future work is needed to discover and understand new catalyst materials for oxidative reforming of NH_3_. There is much room to further develop alloy catalysts and suitable support materials that enable desirable alloy formation and substrate binding. Opportunities exist to pair electron-donating supports or ligands to these active metals to facilitate nitrogen desorption steps in oxidation mechanisms.

### Photocatalytic reforming

Photocatalytic reforming of ammonia shows great promise as a sustainable pathway to produce ammonia-derived hydrogen. There are five processes that underlie photocatalytic ammonia reforming, and to photocatalysis in general. These processes are: (1) generation and (2) separation of photoelectron–hole pairs, (3) absorption of light, (4) adsorption/desorption of reactants and products and (5) redox reactions on the surface of the photocatalysts.^88^ In recent years, significant advances have been made in the design of heterogeneous photocatalysts for reforming applications, and we highlight some of these advances as well as potential opportunities in the field.

In ammonia photocatalytic reforming, a catalytically active metal is typically paired with a photoactive support material; metal nanoparticles are often used to allow for better charge separation.^89^ Catalytic metals often feature noble metals such as Pt, Pd or Ag, which have significant costs associated with them. Support materials include TiO_2_, g-C_3_N_4_, Ag_3_PO_4_ and ZnO as examples. Dopants are often utilized to adjust the band gap to shift the absorption into the visible region.

One of the first reports on using TiO_2_ as a photocatalyst for NH_3_ oxidation to produce N_2_ was published in 1979 by Mozzanega *et al.* They used TiO_2_ only as a photocatalyst.^90^ An early study by Taguchi and Okuhara^91^ investigated a range of metal nanoparticles such as Pd, Pt, Cu, Ni, Co, Rh and Ru supported on P-25 TiO_2_ for oxidation of aqueous ammonia. They observed that the N_2_ production rates trended Pt/TiO_2_ ≫ Ru/TiO_2_ > Pd/TiO_2_ > Rh/TiO_2_. Altomare and Selli^89^ showed that Ag/TiO_2_ had higher NH_3_ conversion than Pt/TiO_2_ and Pd/TiO_2_ but was not as selective to N_2_ as Pd/TiO_2_. There have been several successful recent efforts to use more abundant and less expensive metals in photocatalytic materials for ammonia oxidation. Utsunomiya *et al.*^92^ tested the following metals supported on TiO_2_: V, Cr, Ni, Cu, Co, Fe and Mn. Of these metals, Ni/TiO_2_ exhibited the best N_2_ selectivity in aqueous solutions. In a departure from using TiO_2_-based materials, Chen *et al.* investigate the use of silver phosphate (Ag_3_PO_4_) as a photocatalyst,^93^ which advantageously features the absorption of visible light wavelengths. They compared the activity of Ag_3_PO_4_ to that of N-doped TiO_2_ and g-C_3_N_4_ photocatalysts. Ag_3_PO_4_ demonstrated activity for NH_3_ reforming under visible light illumination at ambient conditions. While N-doped TiO_2_ and g-C_3_N_4_ are known to be active as photocatalysts in the visible range, they were not active for NH_3_ reforming. The authors correlated the formation of ˙OH radicals by Ag_3_PO_4_ with the activity in NH_3_ reforming, as ˙OH radicals were not observed with the other two materials.

There is still much more room for progress in the development of NH_3_ reforming photocatalysts under oxidative and non-oxidative conditions. Shifting the light absorption range from UV to visible or even near-infrared ranges is desirable and should be further investigated to utilize solar energy inputs more efficiently in such systems. Additionally, utilizing bimetallic nanoparticles such as AuPt nanoparticles^94^ could provide additional activity and selectivity beyond their monometallic counterparts and should be further investigated in the future.

## Reformer reaction engineering and scaling considerations

Beyond catalyst fundamentals, myriad reactor characteristics influence the performance of a given ammonia reforming catalyst. In this section, as a complement to the present literature emphasis on catalyst discovery and evaluation, we discuss important meso- and macroscale aspects of technical catalysts, reactor designs and integration strategies to motivate device development for various end uses. The four main topics of focus are (1) technical catalyst properties, (2) hydrogen product quality, (3) energy (*i.e.*, heat and light) management and (4) exhaust management.

### Technical catalyst properties

Technical catalysts often vary significantly from laboratory research catalysts in physical form, chemical composition and acceptable performance criteria.^95^ For ammonia reforming, some primary considerations of a candidate technical catalyst are its metal identity, metal loading, structural formulation and stability.

First, trends in sustainable metal sourcing and global availability urge the substitution of platinum-group metals (PGM) with more environmentally benign catalytic metals. While ruthenium exhibits good sustainability metrics,^81^ its relatively high price has prohibited industrial adoption for Haber–Bosch synthesis;^96^ the authors predict similar economic constraints for the use of Ru in commercial NH_3_ reforming applications, although catalyst costs for downscaled devices may be acceptable. However, only a limited number of reports detail metals beyond Ru and Pd to facilitate this chemistry (Table 1 and Fig. 1).^23,78,97–102^ It has been hypothesized that N_2_ desorption limits the effective kinetic rates of NH_3_ decomposition on Fe-, Co- and Ni-based catalysts, while other late transition metals such as Cu, Rh, Pd, Ir and Pt are limited by N–H bond scission kinetics;^35^ in all cases, Ru is regarded as a highly active metal for NH_3_ thermal^78^ and oxidative^84,85^ reforming. Similarly, low metal contents generally make for more cost-effective materials, but deactivation phenomena may necessitate supra-stoichiometric loadings to prolong catalyst lifetime (*vide infra*). The authors encourage further research into Earth-abundant, low-cost metals, metal precursors and material preparation methods that yield catalysts with high activity and thermal/oxidative stability for NH_3_ reforming.

The required form factor of a technical catalyst for ammonia reforming is largely defined by the intended application. While chemical manufacturers frequently employ packed bed reactors, such reactor types and catalyst forms are only appropriate in stationary settings. Indeed, extruded catalyst pellets are not amenable to mobile applications (such as on-board NH_3_ reforming on an aircraft, maritime vessel or off-road vehicle), where frequent vibrations and vehicle movement would lead to bed unpacking, pellet attrition and/or entrainment of fines.

Instead, monolith reactors are attractive for mobile environments. Monoliths are constructed from metal or ceramic materials that are washcoated to support a catalyst.^103^ Ubiquitous in automotive exhaust, monoliths feature high mechanical strength, equal flow conditions across all channels, low pressure drop and dynamic, transient operability.^104,105^ However, catalyst loadings per volume tend to remain low compared to traditional packed beds, which is a disadvantage for any kinetically limited process. These features and limitations imply distinct performance attributes compared to laboratory microreactors operated at differential conversion with powdered catalysts. Indeed, reports on NH_3_ reforming that employ monoliths are scant in the literature.^13,106,107^ Plana *et al.* reported Ni/Al_2_O_3_/cordierite monoliths for thermal reforming, exhibiting complete conversion of pure NH_3_ at 600 °C.^106^ Compared to packed bed experiments with <200 μm particles of crushed monolith catalyst or of un-supported Ni/Al_2_O_3_, the intact cordierite-supported Ni catalyst is capable of 10–20% higher NH_3_ conversions even when the monolith is operated at 100 °C lower temperatures.^106^ Clearly, catalyst form factor and construction are critical performance descriptors, suggestive of enhanced mass and/or heat transfer or possible hydrodynamic advantages when NH_3_ conversion is carried out in real devices. Separately, Wang *et al.* demonstrated >99.9% conversion at the same temperature and 1100 sccm NH_3_ feed flowrate over a microfibrous CeO_2_-promoted Ni/Al_2_O_3_ monolith catalyst for 300 h on stream,^107^ whereas Kane *et al.* successfully performed on-board NH_3_ reforming over a FeCrAl monolith-supported 4.7 wt% Ru/Al_2_O_3_ catalyst integrated within a John Deere 6400 tractor.^13^ As a complement to these works, we recommend dedicated research efforts by the ammonia energy community to understand washcoated (*vs.* powdered) catalyst performance and dynamic monolith cycling strategies for on-board NH_3_ reforming. Advances in 3D printing of catalyst supports^108^ and monolith structures^109,110^ are a further avenue of promising research that may help mitigate effective surface area and volume constraints.

Lastly, a key requirement of technical reforming catalysts is their long-term stability and robustness against deactivation. Deactivation of ammonia reforming catalysts is generally not well understood in the literature^47,54,82,98,111^ and is worthy of significant attention from the research community. From a reaction engineering perspective, physical pore fouling, active site poisoning, and/or thermal and mass gradients leading to active site restructuring (especially during dynamic and/or oxidative cycling) are the likely deactivation modes of an ammonia reforming catalyst. Intentional fouling/poisoning with known or predicted feed/recycle impurities, accelerated aging^112^ and extended time-on-stream testing under gradientless conditions^113^ are urgently needed to advance the viability of NH_3_ reforming catalyst candidates for a given application. Practitioners should carefully choose reaction conditions for deactivation studies^114^ that result in incomplete, integral (moderate) conversions in order to observe diminishing catalyst activities over extended spacetimes.

### Hydrogen product quality

The requisite hydrogen purity that must exit a catalytic ammonia reformer is specified by the end use. Indeed, for IC piston engines and turbines, only ∼25–30 vol% H_2_ in unreacted NH_3_ is required to produce a fungible fuel blend that matches target post-combustion enthalpies for stationary power or vehicle propulsion (Scheme 1). These IC uses necessitate only a single suitable reforming catalyst and reactor. Higher purity applications for FCs, however, require more dedicated strategies to isolate and purify H_2_.^82^ Given the equilibrium limitation of the thermal reforming pathway (eqn (1)) and the N_2_ and H_2_O (eqn (2) only) co-products, complete conversion to pure hydrogen is not achievable over any sole catalyst—thus requiring creative intensification strategies to simultaneously generate and purify the desired H_2_ product. Moreover, both IC and FC converters may feed fuels at elevated pressures, requiring auxiliary compressors or catalytic reformers that operate at high pressure despite the unfavorable equilibrium penalty (eqn (1)).

Various configurations of a reforming reactor integrated with a hydrogen separator are appropriate strategies to generate high-purity (>99.999%) or ultra-high-purity (>99.9999%) H_2_ streams for vehicle refueling or distributed FC utility stations. Stationary systems may afford packed bed reactors integrated with downstream and/or interstage sorbent or membrane separators with NH_3_ recycle loops, whereas mobility applications may require intensified, spatially compact catalytic membrane reactors, with the NH_3_ dissociation catalysis and H_2_ removal occurring simultaneously at the same local time and length scales.^82,115^ Pd-based membranes are well-known for selective H_2_ separation,^116,117^ and membrane reactors for NH_3_ reforming have been reported in the literature.^118,119^ One recent study has successfully demonstrated the latter for maritime applications up to 86% supra-equilibrium H_2_ yields at up to 99.998% purity at operating temperatures of ≥425 °C; further, when the permeate was subjected to a vacuum, essentially complete NH_3_ conversion was achieved by rapid reaction rates at 400 °C, well beyond equilibrium reaction limitations.^120^ Additional research is needed to identify additional NH_3_-tolerant membrane, absorbent and adsorbent materials suitable for intensified catalytic reforming strategies.

### Energy management

**Fig. 3:**
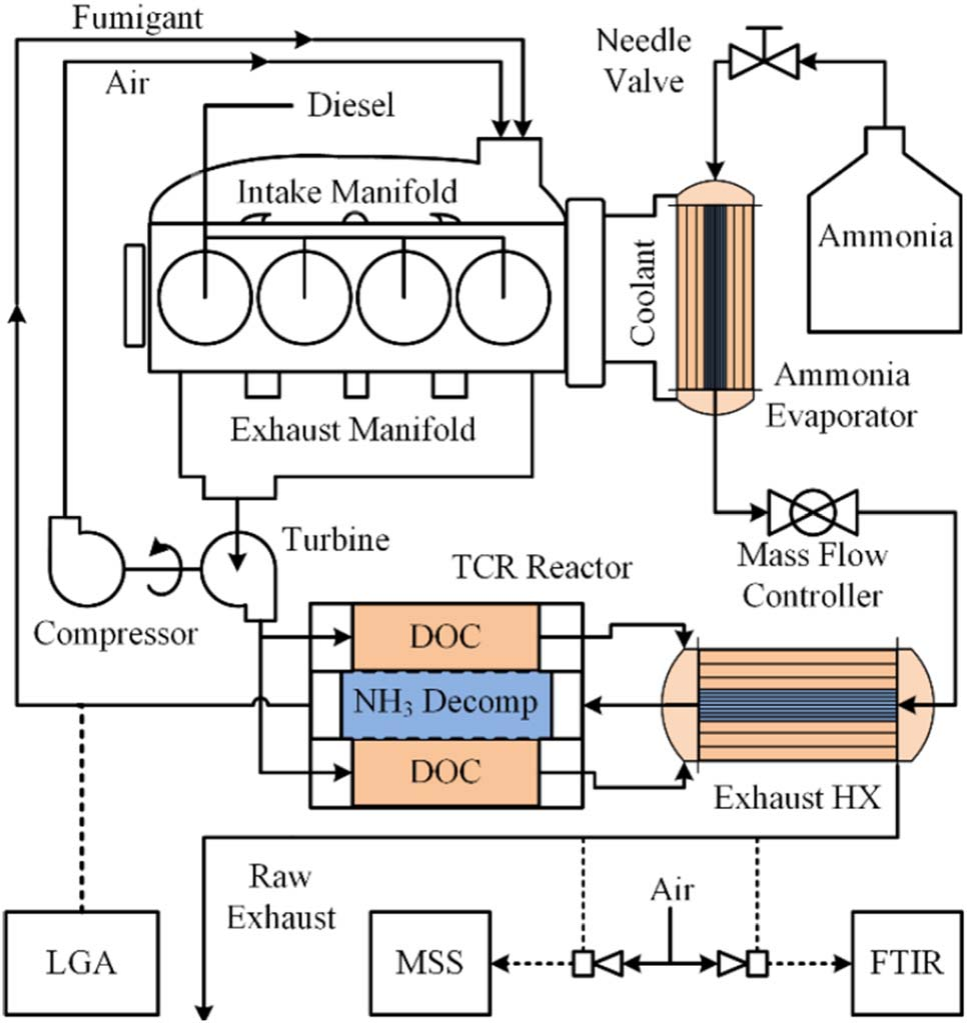
On-board anhydrous NH_3_ thermal reforming reactor and heat exchangers for a diesel/NH_3_ dual-fuel tractor engine and exhaust system.^13^ Reproduced with publisher permission from ref. 13.

Energy-intensive endothermic reactions such as eqn (1) are challenging to implement in any situation, but particularly so on board of aircraft, maritime vessels or off-road vehicles without auxiliary thermal energy generation systems. Separate challenges arise when light (photons) must be introduced to catalytic reactors outside of laboratory settings.^121,122^ Effective recovery and transfer of sensible heat from combustion exhaust streams and/or photons and heat derived from practical light sources are therefore crucial for on-board energy management. Energy balances across monoliths include both axial convection and radial conduction terms, complicating interpretation of experimental results and the development of multiscale models.^105^ Recently, Danilov and Kolb described an autothermal hydrocarbon reforming monolithic reactor using a tanks-in-series model,^123^ which may be applicable to autothermal ammonia reforming systems. Sensible heat recuperation from exhaust gases has been effectively demonstrated on board a John Deere 6400 tractor that employed a 4.7 wt% Ru/Al_2_O_3_ thermal reforming (eqn (1)) catalyst supported on a FeCrAl monolith for a dual-fuel diesel/anhydrous NH_3_ engine (Fig. 3).^13^ Specifically, heat exchanger efficiencies of up to 98% were achieved for NH_3_ flow rates spanning 1.5–12.5 kg h^−1^ for NH_3_ conversions of ∼30–50% across various load cycles. While encouraging for an agricultural tractor, such thermal recuperation schemes may be more challenging to implement for an aircraft or a maritime vessel, where higher mass throughputs and heat transfer rates and efficiencies are expected. Ammonia reformers in these contexts pose a steep design challenge of low mass, compact footprint, high effective surface area, low pressure drop and low thermal latency. Further, spatially compact, highly efficient heat exchanger designs are crucially important for endothermic ammonia reformers (*i.e.*, eqn (1) only).^124^ Finally, light-driven systems are hampered by light attenuation limitations as per the Beer–Lambert Law,^122^ as well as non-uniform light intensity and photon penetration into densified catalyst forms, constraining their adoption in mobility applications.

Alternatively, drawing inspiration from the chemical industry, endothermic reactions are regularly coupled with exothermic reactions to supply sufficient thermal energy *in situ* and/or along reactor vessel walls. Perhaps the most ubiquitous commercial practice of enthalpic coupling is steam methane reforming^125^ (SMR; +206 kJ mol_CH_4__^−1^), which is paired with *in situ* water-gas shift (−41.2 kJ mol_CO_^−1^), *in situ* partial methane oxidation (−35.6 kJ mol_CH_4__^−1^) and/or *ex situ* reactor wall-side natural gas combustion (−804 kJ mol_CH_4__^−1^). Remarkably, SMR has >4× the enthalpy requirement of eqn (1), suggesting that less demanding thermal coupling approaches exist for ammonia reforming. Such intricately coupled reaction engineering strategies enable energy-efficient endothermic or even autothermal^126^ steady-state chemical transformations, as well as minimally energy-intensive reactor start-up operations. Chiuta and Bessarabov reported an autothermal microreactor for NH_3_ decomposition (eqn (1)) that supplied heat *via* exothermic NH_3_ oxy-combustion in alternating, countercurrent catalytic plate channels;^127^ related approaches were modeled previously by Deshmukh and Vlachos.^128^ Ammonia autothermal reforming (NH_3_–ATR) combining eqn (1) and (2) has been demonstrated in laboratory reactors^84,85^ and, promisingly, for a diesel engine employing exhaust gas recirculation (EGR).^129^ In the latter case, an optimal sub-stoichiometric O_2_/NH_3_ feed ratio of 0.04–0.175 was achieved for 2.5–3.2 L min_H_2__^−1^ over a pelleted 2 wt% Ru/Al_2_O_3_ catalyst at a constant NH_3_ feed rate of 3.0 SLM. H_2_ and reformer efficiencies were measured up to 80% and 102%, respectively,^129^ highlighting the viability of on-board NH_3_–ATR strategies for off-road mobility.

Additional research is recommended to understand cold-start and steady-state phenomena at each the catalyst, reformer and systems levels for different modes of heating (heat exchange, autothermality, Joule heating and combinations thereof). Joule heating is particularly attractive for hybrid vehicle systems that employ batteries and/or FC converters, which could in turn supply renewable electrons to reformer monoliths *via* local resistive heating; other creative concepts such as induction heating of electromagnetically active catalysts (*e.g.*, core–shell materials^130^) may also offer promising solutions for next-generation vehicles. Stationary NH_3_ reforming applications offer significantly more latitude to employ Joule heating by renewable electricity, especially if catalysts and downstream H_2_ reservoirs are insensitive to thermal cycling. Finally, non-traditional reactor concepts such as electrochemical reactors,^131^ microreactors^127,132^ and plasma-driven reactors^133^ and catalysts^134^ are also promising approaches for managing thermal energy flows in ammonia reforming. These and other topics are worthy of future investigations.

### Exhaust management

**Scheme 2:**
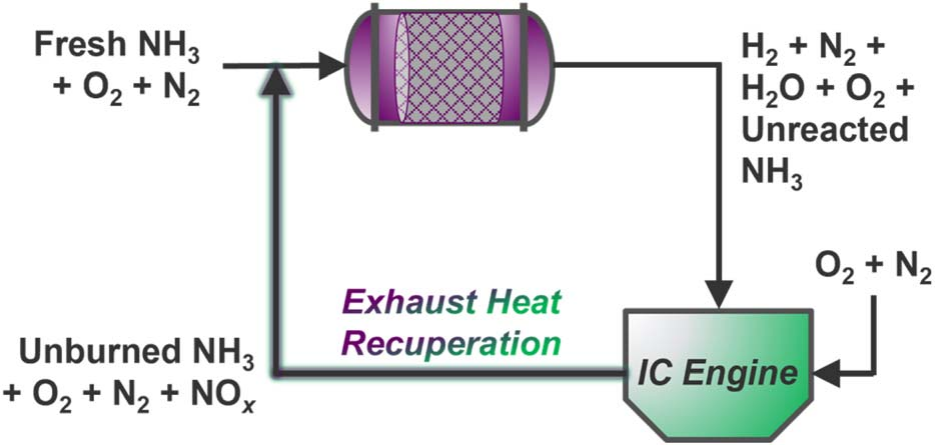
Steady-state intensified NH_3_–ATR and NO_*x*_–EGR concept with continuous mass and heat recycling to an ATR monolith inlet.

Ammonia reforming reactors offer unique integration opportunities with traditional vehicle exhaust systems, many of which already employ NH_3_ reductant (*via* urea solutions) for selective catalytic reduction (SCR) of post-combustion pollutants.^135^ Here, slip streams of ammonia from reformers may be supplied directly to conventional emissions catalysts to reduce harmful NO_*x*_ gases below compliant thresholds; however, it is still unknown whether existing commercial NO_*x*_ reduction catalysts will be suitable for the expectedly more concentrated exhaust resulting from combustion of NH_3_ fuel blends.^4^ Indeed, steady-state EGR has been demonstrated for thermally recuperated NH_3_ reforming using gasoline^136,137^ and diesel^129^ engines, but creative extensions of NO_*x*_ exhaust gas recirculation (NO_*x*_–EGR) could enable intensified thermal/mass recuperation strategies for simultaneous NH_3_ reforming and NO_*x*_ abatement over a dual-functional catalyst bed (Scheme 2). Multiscale and process-level models could aid in determining the dynamic mass and energy balances required for optimal operation, while significant research is yet needed to identify suitable multi-functional catalyst(s) for dual reforming and emissions management. Stationary systems housed in agricultural contexts could further incorporate upstream electrochemical reduction of nitrate contaminants to supply ammonia for on-site or on-board reforming, unlocking inventive concepts in nitrogen atom circularity.

## Summary and outlook

Catalytic ammonia reforming is an important chemical transformation of broad interest to the hydrogen energy and sustainable fuels communities. Whether the NH_3_ is partially reformed for IC engines, turbines or solid oxide FCs, or completely reformed with integrated gas separation for ultra-high-purity H_2_ end uses, the fundamental catalysis and reaction engineering principles underlying such transformations are universal to myriad ammonia and hydrogen energy applications. However, significant knowledge gaps exist to identify and understand catalytic materials capable of dissociating ammonia at low to moderate temperatures, particularly under realistic, dynamic reactor operating conditions. Researchers studying ammonia energy in mobility and/or stationary contexts are recommended to further investigate specific technical hurdles in ammonia reforming, including active site substructures and energetics, catalyst stability, form factor, reactor configuration, and novel strategies for heat management, process intensification and nitrogen atom circularity.

## Author contributions

N. E. T. and M. M. N. conceived of the article topic and structure, and N. E. T., M. M. N., and L. C. C. wrote the article.

## Conflicts of interest

There are no conflicts to declare.

## Supplementary Material
